# Does the organisational model of dementia case management make a difference in satisfaction with case management and caregiver burden? An evaluation study

**DOI:** 10.1186/s12877-016-0237-y

**Published:** 2016-03-09

**Authors:** José M. Peeters, Anne Margriet Pot, Jacomine de Lange, Peter M. Spreeuwenberg, Anneke L. Francke

**Affiliations:** Netherlands Institute for Health Services Research (NIVEL), PO Box 1568, 3500 BN Utrecht, The Netherlands; Netherlands Institute of Mental Health and Addiction, PO Box 725, 3500 AS Utrecht, The Netherlands; Department of Clinical Psychology and EMGO Institute for Health and Care Research, Faculty of Psychology and Education, VU University, Van der Boechorststraat 1, 1081 BT Amsterdam, The Netherlands; Research Centre Innovations in Care, Rotterdam University of Applied Sciences, Rochussenstraat 198, 3015 EK Rotterdam, The Netherlands; Department of Public and Occupational Health and EMGO institute for Health and Care Research, VU University Medical Center, De Boelelaan 1118, 1081 HV Amsterdam, The Netherlands

**Keywords:** Case management, Dementia care, Integrated care, Informal care, Caregiver burden, Satisfaction

## Abstract

**Background:**

In the Netherlands, various organisational models of dementia case management exist. In this study the following four models are distinguished, based on differences in the availability of the service and in the case management function: Model 1: the case management service is available from first dementia symptoms + is always a separate specialist function; Model 2: the case management service is only available after a formal dementia diagnosis + is always a separate specialist function; Model 3: the case management service is available from first dementia symptoms + is often a combined function; Model 4: the case management service is only available after a formal dementia diagnosis + is often a combined function. The objectives of this study are to give insight into whether satisfaction with dementia case management and the development of caregiver burden depend on the organisational model.

**Methods:**

A survey was carried out in regional dementia care networks in the Netherlands among 554 informal carers for people with dementia at the start of case management (response of 85 %), and one year later. Descriptive statistics and multilevel models were used to analyse the data.

**Results:**

The satisfaction with the case manager was high in general (an average of 8.0 within a possible range of 1 to 10), although the caregiver burden did not decrease in the first year after starting with case management. No differences were found between the four organisational models regarding the development of caregiver burden. However, statistically significant differences (*p* < 0.05) were found regarding satisfaction: informal carers in the organisational model where case management is only available after formal diagnosis of dementia and is often a combined function had on average the lowest satisfaction scores. Nevertheless, the satisfaction of informal carers within all organisational models was high (ranging from 7.51 to 8.40 within a range of 1 to 10).

**Conclusions:**

Organisational features of case management seem to make little or no difference to the development in caregiver burden and the satisfaction of informal carers. Future research is needed to explore whether the individual characteristics of the case managers themselves are associated with case management outcomes.

## Background

Dementia is a global health priority. In 2010, about 35.6 million people were living with dementia worldwide, a number that will double every 20 years [1]. Informal carers, often partners or adult daughters, contribute much to the support for the growing number of people with dementia, especially in community settings. Almost all informal carers experience problems in caring for their relative with dementia [2–4]. Cognitive decline, and mood, behavioural and personality changes in the person with dementia are often very challenging and many informal carers experience a high caregiver burden [5, 6]. This may, for instance, be manifested in feelings of having too many responsibilities, having difficulty combining a job and care tasks, and psychological problems [7].

Over the last few years, many initiatives have been taken to support persons with dementia and their informal carers, including case management initiatives. In this paper we define dementia case management as a client-centred strategy to improve the coordination and continuity of the delivery of services for persons with dementia and their informal caregivers [8]. The dementia case manager is the healthcare professional who provides the case management.

Previous research has shown that informal carers and patients are on average satisfied with the support from case managers specialised in dementia [9, 10]. In addition, international literature reviews indicate a reduction in caregiver burden among informal carers thanks to dementia case management [11–13]. However, not all studies included in these reviews showed the same positive outcomes. The ambiguity of the results might be due to the fact that case management can be organised in different ways [14, 15].

In the Netherlands, dementia case management is mainly organised by regional dementia care networks. These networks are formal regional alliances of relevant inpatient and outpatient care providers, such as home-care organisations, nursing homes, elderly care homes, general practitioners and mental-health centres [16]. The main aim of these regional networks is to improve the quality and continuity of dementia care within a region. The Dutch government promotes case management, but gives the regional networks a lot of freedom in how they organise the case management [17]. A national governance structure or standard for case management is therefore lacking. In the period 2010 to 2011, we performed an evaluation study in 13 of the 70 Dutch regional dementia care networks [18], showing the following organisational differences between regions:

- The first organisational difference concerns the start of the service: case management can be available either as soon as the initial symptoms of dementia appear or only after the formal diagnosis of dementia has been made. In those situations where the case management service is available from the initial symptoms, both case management and diagnostics are generally embedded in a multidisciplinary team of one organisation within the regional network. In contrast, in situations where case management support is only available after diagnosis, the case manager generally works in a different setting to the organisation where the diagnostics take place. Professionals and experts do not agree on when the service should be available [8]. Opponents of availability of case management from first dementia symptoms argue that in the early stages it is the general practitioner’s responsibility to provide support and coordinate care. In addition, opponents argue that involving a case manager in an early stage is expensive and not necessary for most clients. In contrast, advocates of the availability of case management from first dementia symptoms contend that patients and their informal carers often suffer from great uncertainty in the initial stages, and may therefore benefit a great deal from early support from a case manager.
- A second difference between organisational models of case management concerns the function of the case manager: case management can either be a separate specialist function or a combined function. In the latter case, the case manager combines the case management function with other roles, for instance also working as a community nurse, a general practice nurse or a social worker. Again there are arguments in favour of and against this solution [9, 10, 14, 17]. A perceived advantage of a separate specialist function is that the case manager can focus completely on dementia care and can therefore develop a lot of relevant knowledge and skills. However, an advantage of a combined function can be that the case manager may often already be providing care to the client and his/her carers (for example as a community nurse or general practice nurse), which may be positive in terms of the continuity of care.

Based on these two distinctive organisational differences described above, we distinguished for the purpose of this study four main organisational models for dementia case management in the Netherlands [18]:

- *Organisational model 1*: case management service is available from the first symptoms of dementia + case management is always a separate specialist function.
- *Organisational model 2*: case management service is only available after a formal diagnosis of dementia + case management is always a separate specialist function.
- *Organisational model 3*: case management service is available from the first symptoms of dementia + case management is often a combined function.
- *Organisational model 4*: case management service is only available after a formal diagnosis of dementia + case management is often a combined function.

Driven by the ongoing discussions in practice and policy about which organisational models for case management are most appropriate in dementia care, the main objective of this paper is to give insight into whether satisfaction with case management and the development of caregiver burden depend on the organisational model of case management.

The research questions addressed are:

1. Is there a difference in caregiver burden between the four case management models?
2. Is there a difference in caregiver satisfaction between the four case management models?

## Methods

### Setting

For this paper we used data from an evaluation study in 13 of the 70 regional networks for dementia care in the Netherlands [18]. The 13 networks signed up to participate in the evaluation study after a call by the researchers via the Dutch Alzheimer’s association. The case managers in all the participating networks had a bachelor degree in nursing or social work, often followed by specialist training in dementia case management. The case load per 1 full time case manager varied between 35 to 70 clients.

Four of the 13 dementia care networks that participated in this study delivered case management according to organisational model 1, three networks according to model 2, three networks according to model 3, and three networks according to organisational model 4. For a description of the features of the different organisational models of case management, see the Introduction.

### Recruitment and sample of informal carers

Informal carers who had started to receive case management within one of the 13 participating regional dementia networks were eligible for inclusion. In the period January-November 2010, about 900 eligible informal carers were asked via their case manager to participate in the study. In principal, during the first case management contact, the case managers asked all the eligible informal carers to participate in the study. Of these 900 informal carers, 648 (72 %) gave permission to the case manager for their name and address to be passed on to the research team. Subsequently, the first author (JP) sent these 648 informal carers a survey questionnaire immediately after the start of case management (T1). The T1 questionnaire was completed by 554 informal carers (response of 85 %). These 554 informal carers received another survey questionnaire one year later (T2); 429 of the group of 554 carers completed and returned the T2 questionnaire (response of 77 %).

Information is provided below on the number of respondents at the two measurement points and in the four organisational models.

- Respondents in *organisational model 1*: T1 *n* = 140; T2 *n* = 118
- Respondents in *organisational model 2*: T1: *n* = 140; T2: *n* = 98
- Respondents in *organisational model 3*: T1: *n* = 138; T2: *n* = 107
- Respondents in *organisational model 4:* T1: *n* = 126; T2: *n* = 106

### Instruments

Satisfaction with case management was measured using the general satisfaction rating item in the ‘Satisfaction with case management questionnaire’. The content validity and comprehensibility and internal consistency (Cronbach’ alpha = .94) of this Dutch-language questionnaire for measuring satisfaction was previously tested and established by De Lange & Pot [19]. The general satisfaction rating item is: ‘We would like to receive your evaluation score of the case manager. Tick here to give a score between 1 and 10′ (the respondent had to choose between 10 numbers, ranging between the worst score of 1 and the best score of 10). This item was only measured at T2, since at T1 the informal carers had not yet had any experience with case management.

To measure the self-perceived informal caregiver burden at T1 and T2, we used *EDIZ*, a 9-item Dutch-language measurement instrument using a Rasch 5-point-scale (‘no!’ – ‘no’ – ‘more or less’ – ‘yes’ – ‘yes!’) that has satisfactory validity and reliability [7, 20]. Earlier research revealed that caregiver burden measured with EDIZ assesses one dimension ranging from little burden to a heavy burden [20].

The answers of informal carers to the nine EDIZ items were coded as dichotomies. In accordance with the EDIZ instructions, the answer categories ‘no!’, ‘no’ and ‘more or less’ were recoded as ‘0’ (for any level of disagreement) and the answer categories ‘yes’ and ‘yes!’ were recoded as ‘1’ (for any level of agreement). The sum scale scores for the nine EDIZ items were computed and ranged from 0 (no burden) to 9 (heavy burden). Descriptive statistics (frequencies and percentages) were used to analyse the EDIZ scores, as well as the satisfaction scores and background characteristics.

### Analysis

The statistical package STATA version 12.0 was used for all descriptive statistics, such as background characteristics of the respondents and informal carers satisfaction with case management in the whole group (see research question 1; [21]). In addition, to answer research question 2 and 3, multilevel modelling (performed with the statistical package MLwiN version 2.02 [22]) was used to test differences between the four case management organisational models thus between the groups (using the chi-square test; *p* <0.05) regarding (1) caregiver satisfaction with case management as a dependent variable at T2 and (2) the differences between T1 and T2 in caregiver burden as a dependent variable.

For the caregiver satisfaction we modelled four indicator variables, for each organisational model (the intercept was omitted from the model). The adjusted mean (with standard error) was estimated within these analyses for each organisational model. The means were adjusted for the covariates and the cluster sampling. The covariates are ‘the relationship of the informal carer to the person with dementia’, ‘informal carers’ perceived health’ and ‘the duration of the informal caregiving’. Next the means between the four organisational models (two at a time) were tested if they differed.

For the caregiver burden, for every organisational model we estimated two means for T1 and T2. Next we tested if the difference between T1 and T2 from one organisational model was equal to the difference between T1 and T2 from another organisational model. Since we compared two differences, potential differences at T1 were controlled. And these means were again adjusted for the covariates, sampling clustering and the repeated nature of the measurements.

Multilevel models are particularly appropriate for research designs where data for participants are organised at more than one level (‘nested’ data). We used multilevel techniques to allow for the fact that respondents (informal carers) were ‘nested’ within the regional dementia networks and to allow for the fact that caregiver burden was measured repeatedly: at T1 (start of case management) and at T2 (one year after the start of case management). In the final multilevel model we added covariates for the informal carers’ background characteristics, namely 1) the relationship of the informal carer to the person with dementia (e.g. spouse, son/son-in-law or daughter/daughter-in-law), 2) informal carers’ perceived health (good, moderate or poor perceived health) and 3) the duration of the informal caregiving (less than one year or one year or more) (see Table 1). The decision to add these background characteristics to the multilevel model was based on the results about the significance of univariate analyses.

**Table 1:** Respondents’ background characteristics (informal caregivers)

Measurement % (n)	Model 1	Model 2	Model 3	Model 4
*Gender*	At start of case management (T1)	At start of case management (T1)	At start of case management (T1)	At start of case management (T1)
	% (*n* = 140)	% (*n* = 140)	% (*n* = 138)	% (*n* = 126)
Men	23 %	24 %	25 %	30 %
Women	77 %	76 %	75 %	70 %
*Age*				
Younger than 55	38 %	34 %	32 %	30 %
55–75	38 %	38 %	37 %	39 %
75 or older	24 %	28 %	31 %	31 %
Average age (range)	61.1 (35–88 years)	63.0 (30–88 years)	64.3 (33–87 years)	64.6 (27–92 years)
*Relationship to person with dementia*				
Partner	40 %	48 %	43 %	51 %
Daughter(−in-law)/Son(−in-law)	54 %	46 %	47 %	42 %
Brother/sister/other relative	3 %	3 %	6 %	4 %
Friend, acquaintance, neighbour	3 %	3 %	4 %	3 %
*Perceived health*				
Excellent/very good/good	65 %	66 %	67 %	62 %
Moderate	34 %	31 %	30 %	35 %
Poor	1 %	3 %	3 %	3 %
*Duration of informal care*				
Less than 1 year	23 %	24 %	38 %	37 %
1 to 3 years	40 %	47 %	41 %	37 %
3 to 5 years	23 %	15 %	13 %	13 %
5 years or longer	14 %	14 %	8 %	13 %

The number of personal face-to-face contacts with the case manager of the total group varied: in this study, 39 % of the informal carers had contact with the case manager three times or less in the first year after the start of case management (not in table). Nearly half of the informal carers (48 %) had had four to ten face-to-face contacts with the case manager, and 13 % of the informal carers had had more face-to-face contacts with the case manager during the first year of case management (not in table). Face-to-face contacts with the case manager (mainly at the home of the person with dementia) were often combined with e-mail or telephone contacts ‘as needed’

### Ethical considerations

An information letter about the aim of the study was sent together with the T1 questionnaire to all participating carers. The letter also mentioned that study participation (i.e. completion of the survey questionnaire) was voluntary and had no consequences for the case management service they received. The responses to the questionnaire were stored and analysed anonymously, in accordance with the Dutch Personal Data Protection Act [23]. Further ethical approval of this study was not required under the applicable Dutch legislation [24], since all participants were competent individuals and this survey study did not involve any interventions or treatments.

## Results

### Background characteristics

Table 1 shows that in the first measurement (T1), about seventy percent to three-quarters of the respondents of the four organisational models were women. The mean age of the respondents varied between 61.1 to 64.6 years (within an age range of 27 to 92). About forty percent to half of the informal carers were the partner of the person with dementia and the other half were mainly daughters/daughters-in-law or sons/sons-in-law. More than sixty percent of the informal carers perceived their heath as excellent/very good/good. Four out of ten carers had looked after their partner or relative for one to three years. There were no significant differences between the four organisational models at T1. Furthermore, there were no significant differences between T1 and T2 in the background characteristics listed in Table 1, which makes sense as there was little drop-out between the two measurement moments.

### Satisfaction with the case manager

The satisfaction with the case manager was high in general (an average of 8.0 for the total group within a possible range of 1 to 10), indicating that carers value the support of the case manager. Of all informal carers, only 5 % gave a low mark (a score of 5 or lower; not in table).

**Fig. 1:**
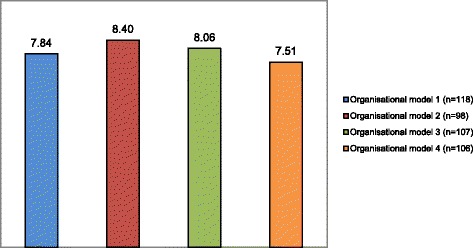
Satisfaction with the case manager, 1 year after start of case management

There are only slight differences between the four organisational models regarding informal caregivers’ satisfaction (see Fig. 1). After statistical correction for background characteristics (see Analyses section), organisational model 2 differs significantly from organisational model 4 (chi-square = 3.84; *p* < 0.05), and organisational model 3 differs significantly from organisational model 4 (chi-square = 10.07; *p* < 0.05). These differences show that informal caregivers who receive case management within organisational models 2 and 3 are more satisfied with case management than caregivers who receive case management within organisational model 4 (see Fig. 1 below; a description is included of the organisational models’ features).

### Caregiver burden

The average total score on the EDIZ scale (the scale used to measure caregiver burden) for the four organisational models was moderately high at the start of case management, namely between 3.6 and 4.1 within a range from 0 (no burden) to 9 (heavy burden).

**Fig. 2:**
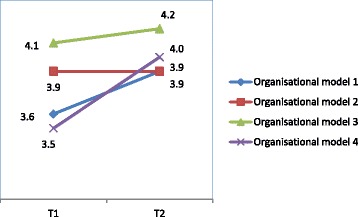
Average perceived burden, at the start of case management and after one year

One year after the start of case management (T2) the total score had increased slightly (from 3.9 to 4.2, see Table 2), however these differences between the four organisational models of case management for the average total score on the EDIZ-scale are not statistically significant (see Fig. 2).

**Table 2:** Perceived caregiver burden (EDIZ)

Measurement (n)	Model 1		Model 2		Model 3		Model 4	
*Average (range, sd)*	At start of case management (T1) (*n* = 116)	1 year after start of case management (T2) (*n* = 48)	At start of case management (T1) (*n* = 121)	1 year after start of case management (T2) (*n* = 50)	At start of case management (T1) (*n* = 119)	1 year after start of case Management (T2) (*n* = 48)	At start of case management (T1) (*n* = 108	1 year after start of case management (T2) (*n* = 59)
My relative’s situation gives me hardly any room to live my own life	3.1 (1–5; 0.09)	3.1 (1–5; 0.13)	3.3 (1–5; 0.09)	3.2 (1–5; 0.13)	3.3 (1–5; 0.09)	3.4 (1–5; 0.13)	3.1 (1–5; 0.09)	3.1 (1–5; 0.12)
It is not easy to combine the responsibility for my relative and the responsibility for my work or family	3.1 (1–5; 0.09)	3.3 (1–5; 0.14)	3.2 (1–5; 0.09)	3.4 (1–5; 0.14)	3.4 (1–5; 0.09)	3.3 (1–5; 0.14)	3.3 (1–5; 0.09)	3.4 (1–5; 0.1.3)
I am letting down other people because of my involvement with my relative	3.7 (1–5; 0.08)	3.9 (1–5; 0.11)	3.7 (1–5; 0.08)	3.6 (1–5; 0.11)	3.9 (1–5; 0.08)	3.8 (1–5; 0.11)	3.7 (1–5; 0.09)	3.7 (1–5; 0.11)
I always have to be prepared for my relative	2.8 (1–5; 0.09)	2.9 (1–5; 0.13)	2.9 (1–5; 0.09)	3.0 (1–5; 0.12)	3.0 (1–5; 0.09)	3.0 (1–5; 0.12)	(1–5; 0.09)	2.6 (1–5; 0.11)
My independence is at stake	3.4 (1–5; 0.08)	3.5 (1–5; 0.12)	3.5 (1–5; 0.08)	3.4 (1–5; 0.12)	3.7 (1–5; 0.08)	3.8 (1–5; 0.12)	3.4 (1–5; 0.09)	3.5 (1–5; 0.12)
My relative’s situation needs my constant attention	2.7 (1–5; 0.09)	2.6 (1–5; 0.12)	2.7 (1–5; 0.08)	2.9 (1–5; 0.11)	2.8 (1–5; 0.09)	2.7 (1–5; 0.12)	2.5 (1–5; 0.09)	2.6 (1–5; 0.12)
My involvement with my relative causes conflicts at home and/or at my work*	3.9 (1–5; 0.08)	4.1 (1–5; 0.11)	3.9 (1–5; 0.08)	4.0 (1–5; 0.11)	4.1 (1–5; 0,08)	3.9 (1–5; 0.11)	3.8 (1–5; 0.08)	3.9 (1–5; 0.11)
My relative’s situation is never out of my mind	2.5 (1–5; 0.11)	2.5 (1–5; 0.16)	2.7 (1–5; 0.12)	2.7 (1–5; 0.16)	2.6 (1–5; 0.12)	2.7 (1–5; 0.16)	2.6 (1–5; 0.12)	2.5 (1–5; 0.15)
My relative’s situation puts me under a lot of pressure	3.0 (1–5; 0.09)	3.1 (1–5; 0.12)	3.1 (−15; 0.09)	3.1 (1–5; 0.12)	3.2 (1–5; 0.09)	3.2 (1–5; 0.12)	3.0 (1–5; 0.09)	3.1 (1–5; 0.11)
Total score for EDIZ**	3.6 (0–9; 0.23)	3.9 (0–9; 0.31)	3.9 (0–9; 0.23)	3.9 (0–9; 0.30)	4.1 (0–9; 0.23)	4.2 (0–9; 0.31)	3.5 (0–9; 0.24)	4.0 (0–9; 0.29)

* Differences between the score of T1 and T2 between model 1 and model 3 are statistically significant (chi-square =5.28, *p* < 0.05)

** Differences between the scores of T1 and T2 are not statistically significant (chi-square, *p* < 0.05)

The covariates are ‘the relationship of the informal carer to the person with dementia’, ‘informal carers’ perceived health’ and ‘the duration of the informal caregiving’.

Looking at the individual items, we see that informal carers gave high scores (i.e. the most unfavourable scores) for the EDIZ items ‘I am letting down other people because of my involvement with my relative’, ‘My involvement with my relative causes conflicts at home and/or at my work’ and ‘My relative’s situation puts me under a lot of pressure’ (see Table 2).

## Discussion

### Main findings and reflections

The satisfaction of the informal carers with case management as offered by the regional networks in the Netherlands is generally high. Informal carers appreciate the support of the case manager. This is in line with previous research, which also found high levels of satisfaction with dementia case management [9, 19].

We also found that the perceived caregiver burden is moderately high on average (a score of 4.2 within a range of 0 to 9, from no burden to a heavy burden). Elements of caregiver burden concern feelings that the independence is at stake, that the relative’s situation requires continuous attention and that the caregiver can never let go of the situation. These aspects of caregiver burden have also been shown in earlier research [25].

Despite the satisfaction of informal carers with case management, the burden experienced by informal carers did not change in the first year after they started with case management. The result that caregiver burden does not decrease after the start of case management does not, however, necessarily mean that case management has no influence on caregiver burden; dementia is a progressive disease, and a further increase in caregiver burden can be expected anyway. We cannot draw hard conclusions about the effects on caregiver burden because there was no control group. However, our main objective was not to look for overall effects but to find out whether case management outcomes are associated with organisational features of case management. Our findings indicate that it does not make a difference for caregiver burden whether the case management service is available from the appearance of the first symptoms or only after the formal diagnosis of dementia, or whether the case manager has a separate specialist function or a combined function in combination with other roles.

However, after statistical correction for background characteristics of the informal carers, we did find small, statistically significant differences between the organisational models regarding satisfaction. Informal carers in the organisational model where case management is only available after a formal dementia diagnosis and is often a combined function have on average the lowest satisfaction scores. Nevertheless, the satisfaction of informal carers within all four organisational models is high (ranging from 7.51 to 8.40 within a range of 1 to 10). Because of the fact that the differences between the organisational models regarding satisfaction are small, we must be cautious in drawing conclusions. Caution is also needed because both in practice and in policy-making, discussions are ongoing about whether case management should offered by a case manager with a separate specialist function or can also be offered by, for instance, community nurses with a combined function. The Dutch government currently aims at giving generalist community nurses a pivotal role in the care for people resident at home [26]. The governmental healthcare policy has resulted in a trend whereby case management as a separate specialist function is becoming less common in some regional dementia care networks while case management as a part-time, combined function for community nurses has become more common [27]. Based on our findings, we cannot draw hard conclusions about whether or not this is an adverse development. Still, another recent Dutch study points in the direction that having case management organised as a separate specialist function, embedded in a multidisciplinary team, and already available before diagnosis is most beneficial for clients as the case managers are then better able to prevent crises [28].

That we could not find many differences between organisational models may also be related to the fact that organisational differences may not be that large in practice. For instance, the availability of the case management service from first dementia symptoms does not imply that all informal carers already receive case management in the pre-diagnosis phase. In our study we found that in the two organisational models where the case management service was available from the first symptoms (models 1 and 3), case management actually started in the pre-diagnosis stage in only approximately 25 to 30 % of cases according to the carers. This might mean that the actual differences between organisational models and also the differences between informal carers involved are not very large. However, after one year the percentages of clients without a formal dementia diagnosis have decreased to 13 and 7 %. Hence the population in the four organisational models is largely comparable regarding their cognitive impairment.

Individual characteristics of the case manager rather than organisational features might have more influence on care satisfaction and caregiver burden: for instance, factors such as the case managers’ experience, competence and expertise, collaboration skills and style of communication (affective or instrumental). The results of a recent systematic mixed studies review shows that effective case management requires case managers with effective communication skills who are able to communicate well with other healthcare professionals [29]. Communication and collaboration skills are not organisational features but are related to competencies of individual case managers. So far, the influence of case managers’ individual characteristics has been neglected in research on dementia case management, and this requires further research.

### Strengths and limitations

The four organisational models are created for the purpose of this study, for a better understanding of which organisational features are associated with caregiver burden and satisfaction. Research into case management is still in its infancy and often neglects the fact that case management can be organised in many different ways. Hence strength of this study is that we took account of the possible relationship between different case management organisational models and outcomes.

However, a limitation is that we used a non-random sample of 13 regional care networks that were keen to participate and spontaneously applied. Possibly this may have resulted in an over-representation of well-functioning regional networks and may have influenced the results. So the results of this study, e.g. regarding the satisfaction of informal carers with case management, may not be generalisable in all respects to other networks or settings where case management is being provided

A final limitation concerns the fact that we do not have specific details about the patients in the four organisational models regarding their functional or other impairments that might contribute to carer burden and that might also be related to how satisfied carers are with case management.

## Conclusions

Satisfaction with case management provided by Dutch regional dementia networks is high, although the caregiver burden does not significantly decrease in the first year after the start with case management. How case management is organised seems to make no difference to the development in caregiver burden and little difference to the satisfaction of informal carers. Future research is needed to explore whether characteristics of the individual case manager, such as communication style, are associated with case management outcomes.
